# HIV Protease Cleavage of Procaspase 8 is Necessary for Death of HIV-Infected Cells

**DOI:** 10.2174/1874357900802010001

**Published:** 2008-01-22

**Authors:** Zilin Nie, Gary D Bren, Stacey A Rizza, Andrew D Badley

**Affiliations:** 1 Division of Infectious Diseases, Mayo Clinic College of Medicine, Rochester, MN 55905, USA; 2 Program in Translational Immunovirology and Biodefense, Mayo Clinic College of Medicine, Rochester, MN 55905, USA

## Abstract

Numerous host and viral factors are capable of causing death of HIV infected cells, uninfected bystander cells, or both. We assessed the relevance of HIV protease in infected cell killing by mutating its obligate substrate for death, procaspase 8. VSV pseudotyped HIV infection of cells expressing WT caspase 8 resulted in apoptotic cell death and generation of the HIV protease specific cleavage product of procaspase 8, casp8p41. Conversely, both cell death and casp8p41 production were inhibited in cells expressing procaspase 8 engineered to be resistant to HIV protease cleavage. Lymph nodes from HIV-infected patients with ongoing viral replication also selectively expressed casp8p41, which colocalized with both infected and apoptotic cells. HIV protease cleavage of procaspase 8 appears to be a necessary event for infected cell killing, which is responsible for infected cell death within lymphoid tissues from HIV-infected patients.

## INTRODUCTION

Numerous factors associated with HIV infection are capable of causing cell death. Broadly, these can be categorized as viral proteins capable of initiating or modifying cell death pathways including Tat, env, Vpr and protease or host factors which are modified in a manner which favors cell death including Fas/Fas ligand, TNF/TNF receptor 1, and/or TRAIL/TRAIL receptor 1 or 2 (reviewed in [1]). Both cells that are directly infected by HIV as well as neighboring uninfected cells die following HIV infection. Potential stimuli which initiate uninfected bystander cell death include soluble Tat, env, and Vpr or death-inducing ligands produced by infected cells acting in trans upon uninfected targets. In reality, it is likely that all of these stimuli cooperatively contribute to the cumulative bystander cell death.

Unlike the situation with uninfected cell death, the molecular stimuli responsible for infected cell death are even less well understood. Such death has been variably described as being lytic or direct cytotoxicity, although the majority of the investigations conclude that infected cells die through apoptosis as evidenced by caspase activation, TUNEL positivity, PARP cleavage, etc (reviewed in [2]). However, even this conclusion remains controversial due to a series of findings where cell lines deficient in selected apoptosis regulatory proteins were infected with HIV and analyzed for death. Since cell death still occurred in cells deficient in RIP, procaspase 8, and FADD, the authors concluded that such death must be necrotic with apoptotic features [3]. It is noteworthy that even under the conditions examined, 10-15% apoptosis was observed, and therefore the authors acknowledged that HIV infection may directly cause loss of mitochondrial transmembrane potential and release of apoptogenic factors, similar to what has subsequently been described for HIV Vpr [4]. In addition, these authors acknowledged that HIV infection might also have a direct effect on caspases, consistent with the known effect of HIV protease on procaspase 8 [5, 6]. Indeed, further evidence of a direct role for apoptosis, but not necrosis in infected cell death is found using the pancaspase inhibitor Z-VAD FMK, which significantly reduces infected cell death [7], and correlations between rates of HIV disease progression and levels of HIV induced apoptosis [8- 10].

HIV protease (PR), unlike other HIV proteins, can only cause death of infected, but not uninfected cells. The substrate specificity of PR is sufficiently degenerate that HIV protease activity which is present within the cytosol of infected cells causes cleavage of a wide variety of host proteins including actin, laminin, Bcl-2, e1F4G, and procaspase 8 (reviewed in [11]). Of these, procaspase 8 is necessary for protease induced cell death since its absence abrogates HIV protease induced mitochondrial depolarization and nuclear fragmentation [5]. The relevance of HIV protease to infected cell killing however is unknown since conflicting data exist concerning the involvement of protease in infected cell death. Heterologous expression of the HIV genome with stop mutations introduced throughout the open reading frames of each HIV protein suggest that protease expression is dispensable for HIV-induced killing, yet in those experiments other HIV proteins, which are known to independently kill, were being overexpressed simultaneously [12]. Other data demonstrating HIV protease-specific cleavage fragments of procaspase 8, which co-localize with both infected and dying cells, argue a more causal role for HIV protease in infected cell death [6]. We have previously shown that HIV protease alone induces cell death through procaspase 8 cleavage producing Casp8p41. The purpose of the current study was to determine whether protease cleavage of Caspase 8 occurs during acute T cell infection and whether this contributes to the overall killing of T cells directly infected with HIV.

## MATERIALS AND METHODS

### Mutagenesis

Plasmid pcDNA3Flag-caspase8-Myc [5] harboring Arg355Asn356 substitution for Phe355Phe356 was created by site-directed mutation using Quickchange Site-directed Mutagenesis Kit (Strategene).

### Induction of Apoptosis

I9.2 cells or I9.2 cells reconstituted with either WT procaspase 8 or procaspase 8 containing the RN mutations at positions 355, 356 (10^6^) were treated with 10 µM campothecin, 100 ng/ml *SuperKiller*TRAIL^TM ^(Axxora), 250 ng/ml anti-Fas antibody CH-11 (Upstate Cell Signaling Solutions), 50 ng/ml TNF-α (R&D Systems) plus 5 µg/ml cycloheximide, 2 µM HIV Tat (NIH AIDS Research and Reference Reagent Program), 1 µg/ml of HIV gp120 (ImmunoDiagnostic), or vehicle controls for 8 h at 37°C. For Vpr-induced apoptosis, cells were incubated with isotonic glucose-Hepes buffer (2.4% glucose, 13 mM Hepes, 68 mM NaCl, 1.3 mM KCl, 4 mM Na_2_HPO_4_, and 0.7 mM KH_2_PO_4_, pH 7.2) alone or containing 10 µM of Vpr-derived peptide (amino acids 61-75) (NIH AIDS Research and Reference Reagent Program) for 4 h at 37°C. After the incubation, cells were washed in PBS and incubated overnight at 37°C.

### Electroporation

10^7^ of each stable I9.2 clone was transfected with 10 ug of HIV protease gene cloned into pEYFP-C1 or vector control using a BTX Electro Square Porator T820 (320 volts, 20 msec) in 0.3 mls serum free media. The cells were washed with media, and then cultured in media with 10% FBS at a concentration of 2 x 10^6^ / ml. After 4 hours, the cells were assayed for viability.

### Measurement of Apoptosis

Apoptosis was measured by annexin V PE and TUNEL staining. In annexin-V staining assay, 1 x 10^6^ cells were harvested, washed, and stained with 2 µl annexin-V PE (BD Pharmingen, San Diego, CA) at 37˚C for 20 minutes. TUNEL staining for detection of apoptosis is according to the manufacturer’s protocol (Roche, Nutley, NJ). Flow cytometry was performed using a FACScan (Becton Dickinson Immunocytometry Systems, San Jose, CA), and analysis was done using CellQuest software.

### VSV-g Infections

VSV-g pseudotyped HIV virus stock were prepared, as we have previously described [4]. Briefly, 5µg HIV-1HxBRUVpr-Env- and 10 µg VSV-g plasmids (the ratio 1:2) were co-transfected into 293 T cells. The pseudotyped HIV viruses were concentrated by centrifugation and titrated by the multinuclear activation of galactosidase indicator (MAGI) assay. The I9.2 cells reconstituted by caspase 8 wt and RN mutant were infected at a MOI of 10. The infected cells (10^4^ cells/per sample) were harvested each 12 hr post-infection for ATP activity assay (ATP-Glo Bioluminometric Cell Viability Assay Kit.

### Casp8p41 Staining

To determine the presence of casp8p41 in apoptotic cells, cells were fixed in 2% paraformaldehyde at 4°C overnight, then permeabilized with PBS +0.1% NP-40 on ice for 2 minutes. Cells were then incubated with the mouse anti-casp8p41 antibody followed, by PE-labeled goat anti-mouse antibody (Becton Dickinson ImmunoCytochemistry) and FITC-labeled rabbit anti-active caspase 3 antibody.

### Patient Samples

Patient samples were obtained from the National Disease Research Interchange (NDRI). Immunohistochemistry on frozen sections was done as previously described [13] using the following antibodies: anti-P24 FITC, anti-active caspase 3 FITC, and anti-caspase8p41 PE, plus goat a Mo IgG-PE. This protocol was reviewed and approved by the Mayo Clinic Institutional Review Board.

## RESULTS

### HIV Protease-Induced T Cell Death Requires WT Procaspase 8

**Fig. (1):**
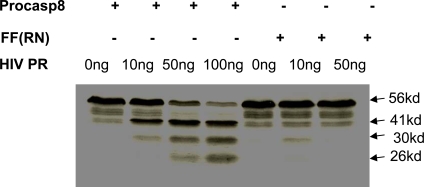
S^35^ wild-type procaspase 8 or procaspase 8 with mutations F355R, F356N were mixed with varying amounts of protease and analyzed by autoradiography for cleavage. Wild-type procaspase 8 incubated with HIV-PR resulted in a 41 kd band that was much less evident in the RN mutant. Results are representative of three independent experiments.

**Fig. (2):**
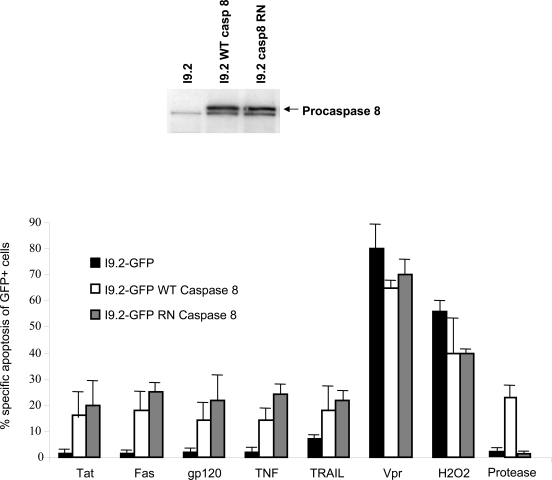
**(A)** Bulk caspase 8 deficient I9.2 cells were stably transfected with either WT procaspase 8 (WT casp8) or procaspase 8 containing the F355R F356N mutant (casp8 RN), and expression of the transgene confirmed by immunoblot for procaspase 8. **(B)** Bulk 19.2 cells stably transfected with GFP, GFP wild-type caspase 8, or GFP RN mutant caspase 8 were treated with Tat, Fas ligation, gp120, TNF, TRAIL, Vpr peptide, H_2_0_2_, or transfected with HIV protease and analyzed for death specifically in the GFP positive cells.

We have previously mapped the site where HIV protease cleaves procaspase 8 as being between phenylalanines at positions 355 and 356, and showed that mutating these residues to Arg (R) and Asn (N), respectively, prevents protease cleavage [6] (Fig. **1**). On the basis of this finding, we have now reconstituted procaspase 8 expression in I9.2 cells, which are normally deficient in procaspase 8 with either WT procaspase 8, or the RN procaspase 8 mutant in order to investigate the role of protease in HIV induced killing (Fig. **2A**). Since procaspase 8 is involved in the apoptotic signaling induced by a breadth of stimuli, we assessed the apoptotic response of I9.2 cells reconstituted by either wild-type procaspase 8 or the RN procaspase 8 mutant. The viability of untreated I9.2 cells over expressing either WT procaspase 8 or RN procaspase 8 mutant was consistently more than 95%. The stimuli we tested included stimuli which had been implicated in cell death associated with HIV infection including Tat, gp120, nef, Vpr, Fas ligation, TNF and TRAIL (Fig. **2B**). Consistent with procaspase 8 signaling being involved in cell death induced by Tat, Fas ligand, TNF, TRAIL and Env, I9.2 cells stably transfected with vector control underwent minimal cell death in response to these stimuli. However, when these cells were reconstituted with either wild-type procaspase 8 or the RN mutant of procaspase 8, death in response to these stimuli was restored, indicating that the RN mutant of procaspase 8 signals normally in response to these death stimuli. Moreover, vector control, WT procaspase 8, and RN procaspase 8 expressing I9.2 cells died equally in response to the caspase 8 independent stimuli, Vpr and H_2_O_2_. However, HIV protease resulted in significant cell death only in I9.2 cells stably expressing WT procaspase 8, but not control I9.2 or I9.2 cells expressing RN procaspase 8. Therefore, cell death induced by HIV protease requires cleavage of procaspase 8. Furthermore, the HIV protease cleavage resistant RN procaspase 8 mutant undergoes death signaling normally in response to all stimuli tested other than HIV protease.

### HIV Induced Cell Death Requires WT Procaspase 8

**Fig. (3):**
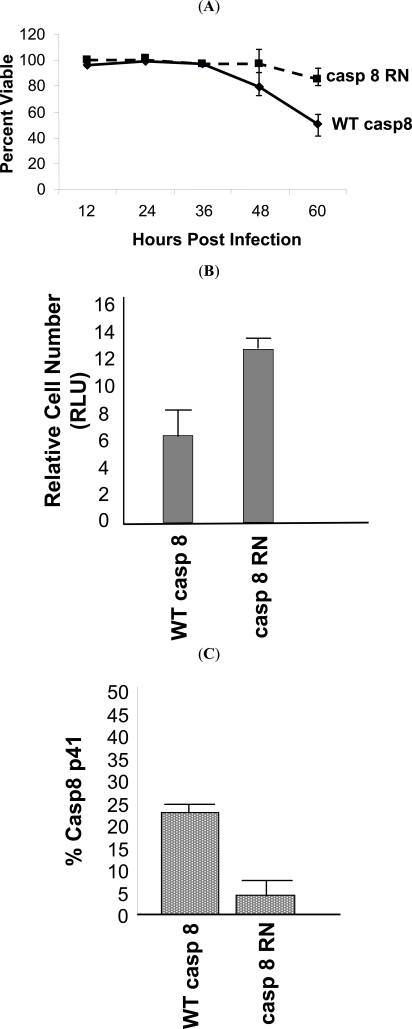
**(A)** I9.2 cells stably transfected with wild type (WT) caspase 8 or caspase 8 with the RN mutation were infected with VSV-G pseudotyped HIV and analyzed for viability as a function of time by trypan blue exclusion. **(B)** The stably transfected I9.2 cells were infected with VSV-G pseudotyped HIV and analyzed for relative cell number 60 hours post-infection by ATP activity. (**C**) Cells harvested 60 hours post-infection were also assessed for casp8p41 content. Results are representative of three independent experiments.

The cleavage of procaspase 8 by HIV protease generates a unique fragment of procaspase 8, which is not produced following other death stimuli, called casp8p41 [6]. Using a monoclonal antibody that specifically recognizes the carboxyl terminus of casp8p41 and does not recognize either full-length procaspase 8 or other caspase 8 processing intermediates, which are produced during apoptosis induced by other stimuli [6], allows assessment of when HIV protease has cleaved procaspase 8. Since I9.2 cells have low level expression of CD4 (data not shown), robust HIV infection is not possible. Therefore, bulk populations of transfected cells were instead infected with VSV-G-pseudotyped HIV. Since HIV protease can only kill cells that express protease intracellularly, (i.e., infected cells), we used a high MOI of 10, in order to infect a maximal proportion of cells. Infected cells were analyzed for viability (Fig. **3A**), clonogenic potential, (Fig. **3B**), and for casp8p41 expression (Fig. **3C**). Infection of WT procaspase 8 expressing I9.2 cells results in high levels of death as determined by active caspase 3 and high expression of casp8p41. As expected, since the procaspase 8 F355R, F356N mutant is cleaved less by HIV-PR (Fig. **1**), infection of the RN procaspase 8 expressing I9.2 cells resulted in less casp8p41 production, and remarkably, less HIV-induced death. At 60 hours post-infection, I9.2 cells reconstituted with WT casp8 were 57% viable, whereas cells reconstituted with casp8 RN were 92% viable (p<0.05). Thus, introduction of a mutation in procaspase 8 alone is sufficient to achieve two endpoints: 1) abrogating the caspase8 cleavage by protease, and 2) reducing HIV-induced death by blocking production of casp8p41.

### Procaspase 8 Cleavage to Casp8p41 Occurs *In Vivo*

**Fig. (4).**
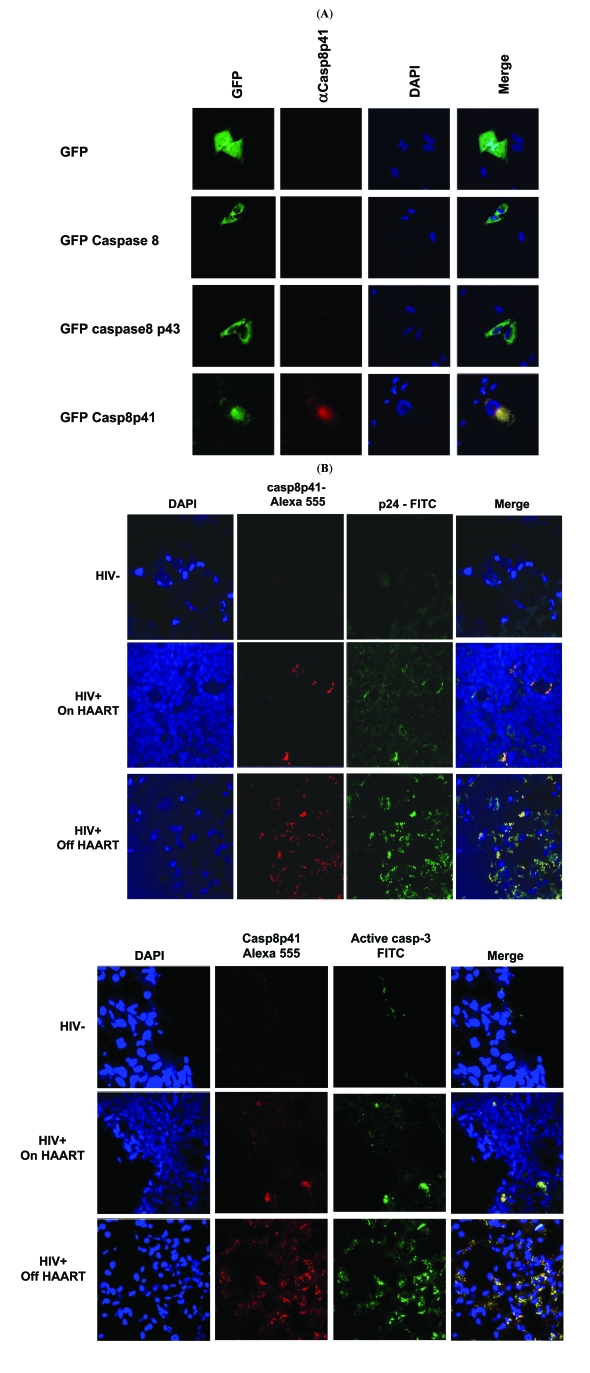
**(A)** Hela cells were transfected with the indicated GFP constructs and stained with anti-casp8p41 PE and DAPI. **(B)** Lymph node sections from HIV negative or HIV infected patients with either suppressed or non-suppressed levels of viral replication were stained with anti-casp8p41 (red) and anti-p24 (green) counterstained with DAPI to identify nuclei and analyzed by confocal microscopy. Results representative of three or more analyses each. **(C)** Lymph node sections from HIV negative or HIV infected patients with either suppressed or non-suppressed levels of viral replication were stained anti-casp8p41 (red) and anti-active caspase 3 (green) and counterstained with DAPI to identify nuclei and analyzed by confocal microscopy. Results representative of three or more analyses each.

Having previously shown that HIV protease kills cells through Casp8p41 [6], and that cell death induced following T cell infection with intact HIV requires procaspase 8 processing by protease, we next assessed whether Casp8p41 occurs in lymphoid tissues from HIV infected patients. Previously, we have shown that Casp8p41 is present in PBL from HIV infected patients predominantly within the CD4^+^ CD27^+^ memory subset, and that Casp8p41 positive cells were apoptotic and contained HIV p24, but that only a small percentage of PBL contained Casp8p41. Since recent data suggest that a significant proportion of T cells that die within lymphoid tissues are physically infected with HIV [14], we chose to examine lymphoid tissues using immunohistochemistry for casp8p41, activated caspase 3 as a marker of apoptosis, and p24 as a marker of HIV infection. First we validated our casp8p41 antibody for use in immunohistochemistry by assessing its immunoreactivity against cells transfected with casp8p41, full length caspase8, or the 43Kd caspase 8 cleavage intermediate (Fig. **4A**). Next, we analyzed at least three different lymph node sections from HIV negative patients, HIV positive patients on suppressive therapy, or HIV positive patients on non-suppressive therapy, for casp8p41, in combination with either HIV-p24 or activated caspase 3. Consistent with our observations in PBL, casp8p41 was detected only in p24 positive infected cells, although not all p24 positive cells were casp8p41 positive (Fig. **4B**). Additionally, all casp8p41 positive cells were active caspase 3 positive (Fig. **4C**), although not all caspase 3 positive cells were casp8p41 positive, indicating that bystander apoptosis is still present, albeit to lesser degrees. The magnitude of casp8p41 staining varied with viral replication; in the HIV negative lymph nodes, no casp8p41 was detected; in HIV-infected patients on suppressive therapy, few casp8p41 positive cells were detected, yet those that were stained strongly positive. Finally, in HIV patients on non-suppressive therapy, an intense casp8p41 staining pattern was observed, with a greater number of positive cells per section. In all cases, casp8p41 positive cells were p24 positive and active caspase 3 positive. Importantly, and distinctly from our findings in PBL where a minority of cells contained casp8p41, in these lymphoid tissues from patients with non-suppressive HIV therapy a majority of cells within follicular areas contain casp8p41.

## DISCUSSION

It has been difficult to determine which signals trigger death in cells infected by HIV, while these cells are dying. We have overcome this obstacle in two ways. Firstly, by reconstituting procaspase 8 deficient cells with either wild-type or a procaspase 8 mutant, and demonstrating that the protease cleavage resistant procaspase 8 cells signal normally in response to all stimuli tested other than protease, thereby allowing the conclusion that if HIV cell death is reduced in these cells, it must be due to reduced protease initiated death. Secondly, using a neoepitope-specific antibody that recognizes only the procaspase 8 fragment produced following protease cleavage [6] to demonstrate that the presence of casp8p41 in patient tissues is a selective marker of these infected cells which are undergoing death.

Understanding that HIV protease can kill cells in a caspase 8 dependant manner [5], *via* a casp8p41 intermediate [6] has allowed us to assess in our current report, whether this process is a necessary event for infected T cell death. Indeed using a VSV pseudotyped infection, abrogation of procaspase 8 cleavage significantly reduces infected cell death. Consequently, death of HIV infected cells occurs in an HIV protease, caspase 8 dependent manner.

Because the majority of apoptotic cells within lymphoid tissues of HIV-infected patients contain casp8p41, this argues that the majority of cell death in lymphoid tissues occurs in infected, but not bystander cells. This contrasts with data that TUNEL-positive apoptotic cells are predominantly bystander, but not productively infected cells [15]. That study used RNA in situ hybridization with five different riboprobes all of >2KB in length to define productive infection [16], which was not observed in those TUNEL positive cells [15]. Our knowledge of the molecular events of apoptosis has increased since that study was published; we now know that during the terminal stages of apoptosis, when DNA is fragmented and TUNEL assays become positive, a variety of apoptotic nucleases are activated, notably DFF40 and endonuclease G [17]. Since endonuclease G has a substrate preference for RNA and ssDNA over dsDNA [17], nascent mRNA transcripts present in apoptotic cell are degraded in parallel with the occurrence of nuclear fragmentation. Therefore, identification of mRNA species of >2KB in cells with fragmented (TUNEL positive) nuclei is an unreliable approach to demonstrate productive HIV infection, since RNA will be degraded at that stage of cell death. Therein lies the difficulty of determining whether infected and apoptotic cells can be identified. Our conclusion that apoptotic cells in lymphoid tissues are predominantly infected and dying by a casp8p41 pathway, but not bystander apoptotic pathways, comes from three complementary observations: 1) casp8p41 is produced only by HIV protease, and therefore is present only in infected cells; 2) casp8p41 colocalizes with HIV p24, and 3) casp8p41 co-localizes with active caspase 3.

## CONCLUSIONS

The contribution of infected cell death relative to uninfected (bystander) cell death during HIV infection remains unknown. Certainly, the HIV proteins, Tat, Nef, Env, and Vpr, all contribute to an apoptosis-prone state and/or initiate uninfected cell death in HIV-infected patients. However, identifying HIV protease generated casp8p41 as a dominant cause of infected cell death *in vitro* and within lymphoid tissues *in vivo*, offers opportunities to study the molecular events that favor infected cell death, and why certain cell types, including macrophage and dendritic cells, do not die following infection.

## ABBREVIATIONS

TAT: = Transactivator
ENV: = Envelope
Vpr: = Viral Protein R
TUNEL: = Terminal UTP Nucleotide End Labeling
PARP: = Poly ADP Ribase Polymerase
FADD: = Fas Associated Death Domain
RIP: = Receptor Interacting Protein
HIV: = Human Immunodeficiency Virus
VSV: = Vesicular Stomatitis Virus
TRAIL: = TNF Related Apoptosis Inducing Ligand
PBL: = Peripheral Blood Lymphocyte
DNA: = Deoxyribonucleic Acid
RNA: = Ribonucleic Acid

## References

[1] Badley AD (2006). Cell Death During HIV Infection.

[2] Kreisberg JF, Doitsh G, Greene WC, Badley AD (2006). Mechanisms of HIV-infected *vs* uninfected T cell killing. Cell Death During HIV Infection.

[3] Bolton DL, Hahn BI, Park EA, Lehnhoff LL, Hornung F, Lenardo MJ (2002). Death of CD4(+) T-cell lines caused by human immunodeficiency virus type 1 does not depend on caspases or apoptosis. J Virol.

[4] Lum JJ, Cohen OJ, Nie Z (2003). Vpr R77Q is associated with long-term nonprogressive HIV infection and impaired induction of apoptosis. J Clin Invest.

[5] Nie Z, Phenix BN, Lum JJ (2002). HIV-1 protease processes procaspase 8 to cause mitochondrial release of cytochrome c, caspase cleavage and nuclear fragmentation. Cell Death Differ.

[6] Nie Z, Bren GD, Vlahakis SR (2007). Human immunodeficiency virus type 1 protease cleaves procaspase 8 *In vivo*. J Virol.

[7] Chinnaiyan AM, Woffendin C, Dixit VM, Nabel GJ (1997). The inhibition of pro-apoptotic ICE-like proteases enhances HIV replication. Nat Med.

[8] Liegler TJ, Yonemoto W, Elbeik T, Vittinghoff E, Buchbinder SP, Greene WC (1998). Diminished spontaneous apoptosis in lymphocytes from human immunodeficiency virus-infected long-term nonprogressors. J Infect Dis.

[9] Franceschi C, Franceschini MG, Boschini A (1997). Phenotypic characteristics and tendency to apoptosis of peripheral blood mononuclear cells from HIV^+^ long term non progressors. Cell Death Differ.

[10] Wasmuth JC, Klein KH, Hackbarth F, Rockstroh JK, Sauerbruch T, Spengler U (2000). Prediction of imminent complications in HIV-1-infected patients by markers of lymphocyte apoptosis. J Acquir Immune Defic Syndr.

[11] Nie Z, Badley AD (2006). HIV Protease and Cell Death. Cell Death During HIV Infection.

[12] Sakai K, Dimas J, Lenardo MJ (2006). The Vif and Vpr accessory proteins independently cause HIV-1-induced T cell cytopathicity and cell cycle arrest. Proc Natl Acad Sci USA.

[13] Badley AD, Dockrell DH, Algeciras A (1998). *In vivo* analysis of Fas/FasL interactions in HIV-infected patients. J Clin Invest.

[14] Brenchley JM, Price DA, Douek DC (2006). HIV disease: fallout from a mucosal catastrophe?. Nat Immunol.

[15] Finkel TH, Tudor-Williams G, Banda NK (1995). Apoptosis occurs predominantly in bystander cells and not in productively infected cells of HIV- and SIV-infected lymph nodes. Nat Med.

[16] Smith PD, Fox CH, Masur H, Winter HS, Alling DW (1994). Quantitative analysis of mononuclear cells expressing human immunodeficiency virus type 1 RNA in esophageal mucosa. J Exp Med.

[17] Widlak P, Garrard WT (2005). Discovery, regulation, and action of the major apoptotic nucleases DFF40/CAD and endonuclease G. J Cell Biochem.

